# Perivascular epithelioid cell tumors (PEComas) of the bone and soft tissue: a Japanese Musculoskeletal Oncology Group (JMOG) multi-institutional study

**DOI:** 10.1007/s00432-023-05114-1

**Published:** 2023-07-20

**Authors:** Yuya Izubuchi, Shunsuke Hamada, Yoshikazu Tanzawa, Ikuo Fujita, Jungo Imanishi, Hirotaka Koyanagi, Akiyoshi Shimatani, Tadashi Komatsubara, Takaaki Tanaka, Mana Fukushima, Yoshiaki Imamura, Takafumi Ueda, Hirotaka Kawano, Akihiko Matsumine

**Affiliations:** 1 Department of Orthopaedics and Rehabilitation Medicine, Unit of Surgery, Division of Medicine, Faculty of Medical Sciences, University of Fukui, 23-3 Matsuokashimoaizuki, Eiheiji-cho, Yoshida-gun, Fukui, 910-1193 Japan; 2 Department of Orthopaedic Surgery, Aichi Cancer Center Hospital, Nagoya, Japan; 3 Department of Orthopaedic Surgery, Surgical Science, Tokai University School of Medicine, Kanagawa, Japan; 4 Department of Orthopaedic Surgery, Hyogo Cancer Center, Akashi, Japan; 5 Department of Orthopaedic Oncology and Surgery, Saitama Medical University International Medical Center, Hidaka, Japan; 6 Department of Orthopaedic Surgery, Tokyo Medical and Dental University, Tokyo, Japan; 7 Department of Orthopedic Surgery, Osaka Metropolitan University Graduate School of Medicine, Osaka, Japan; 8 Department of Musculoskeletal Oncology and Rehabilitation, National Cancer Center Hospital, Tokyo, Japan; 9 Department of Tumor Pathology, Faculty of Medical Sciences, University of Fukui, Fukui, Japan; 10 Division of Diagnostic Pathology/Surgical Pathology, University of Fukui Hospital, Fukui, Japan; 11 Department of Orthopaedic Surgery, National Hospital Organization Osaka National Hospital, Osaka, Japan; 12 Department of Orthopaedic Surgery, Teikyo University School of Medicine, Tokyo, Japan

**Keywords:** PEComa, Soft tissue, Bone, Pathological characteristics, TFE3

## Abstract

**Purpose:**

Perivascular epithelioid cell tumors (PEComas) of the bone and soft tissues are rare mesenchymal neoplasms, some of which are malignant. However, their clinical and pathological characteristics remain unclear. This study was performed to investigate the clinical and pathological characteristics of PEComas in bone and soft tissues by leveraging information from the Japanese Musculoskeletal Oncology Group.

**Methods:**

Nine patients, including four male and five female patients with a median age of 50 years, were retrospectively reviewed. PEComas of the visceral organs, including the uterus and retroperitoneum, were excluded.

**Results:**

Eight tumors arose in the soft tissue and one in the bone, with a mean size of 8.8 cm. Four patients showed local recurrence or distant metastasis. The 1-year survival rate was 78%. Pathologically, eight tumors were classified as malignant and one as having uncertain malignancy potential. Half of the tumors showed high MIB-1 index values of > 30%. Immunohistochemically, the melanocyte marker HMB45 was expressed in 89% of the cases, and muscle-specific markers were expressed only in 30–50% of the cases. Transcription factor binding to IGHM enhancer 3 (TFE3) expression was positive in 100% of the patients. Tumors with high expression of TFE3 were classified as PEComas with malignant potential according to Folpe’s classification.

**Conclusions:**

Bone and soft tissue PEComas may have a higher malignancy potential than other visceral PEComas and are more likely to develop as TFE3-rearranged PEComas.

## Background

Perivascular epithelioid cell tumors (PEComas) are a rare family of mesenchymal tumors (Bonetti et al. 1992) and classified as "benign" or "malignant" by the 2020 WHO classification (WHO 2020). The PEComa family of tumors includes angiomyolipoma, lymphangioleiomyomatosis, and PEComa-NOS (Fadare et al. 2004). Most PEComas are sporadic; however, a small subset is associated with tuberous sclerosis (Bonetti et al. 2001).

PEComas usually have a benign clinical course, but they sometimes show a malignant clinical course that leads to local recurrence and/or distant metastases. Histologically, PEComas show a typical nested architecture and are composed of epithelioid cells with abundant granular eosinophilic or clear cytoplasm as well as round nuclei with small nucleoli (WHO 2020). In previous reports, malignant PEComa showed a large tumor size, a high mitotic rate, and the presence of necrosis and nuclear atypia. Folpe classified cases with two or more worrisome features, including size ≥ 5 cm, high-grade nuclear features, infiltration, necrosis, lymphovascular invasion, and mitotic rates ≥ 1/50 high-power fields, as malignant PEComa (Folpe et al. 2005). Immunohistochemically, PEComas typically express melanocytic markers, such as human melanoma black 45 (HMB45) and melanoma antigen (melan-A), and muscle markers, such as smooth muscle actin (α-SMA), desmin, and caldesmon (Hornick and Fletcher 2006).

Common sites of PEComa occurrence are the uterus, kidney, liver, lung, abdominopelvic soft tissues, gastrointestinal organs, retroperitoneum, and skin (Folpe et al. 2005; Ligel et al. 2008; Hornick and Fletcher 2008; Doyle et al. 2013). PEComas of the bone and soft tissues are very rare, and the literature is restricted to case reports and small case series (Mahera et al. 1997; Kuroda et al. 2000; Diment and Colecchia 2003; Fukunaga 2004; Harris et al. 2004; Folpe et al. 2005; Mai and Belanger 2006; Pikoulis et al. 2007; Osei et al. 2007; Blechet et al. 2007; Boussouga et al. 2008; Argani et al. 2010; Yamashita and Fletcher 2010; Varshney et al. 2011; Alnajar et al. 2018; Harvey et al. 2019; Zhong et al. 2020; Rehman et al. 2021). Consequently, the clinical course, clinicopathological characteristics, and treatment strategies for PEComas of the bone and soft tissues are largely unknown.

This study aimed to investigate the clinical and pathological characteristics of PEComas in bone and soft tissues by leveraging information from the Japanese Musculoskeletal Oncology Group (JMOG).

## Methods

### Patients

Patients with PEComas treated at institutions that are part of the JMOG were included in this study. The five institutions participating in the present study were high-volume centers in the field of sarcoma treatment in Japan. We excluded PEComas involving the gastrointestinal tract, uterus, bladder, retroperitoneum, thoracic cavity and other visceral organs and included those arising in the extremities and intramuscular lesion of the trunk. None of the patients had signs or history of tuberous sclerosis.

Patient information on PEComas was collected from JMOG facilities. For pathological evaluation, unstained tumor samples embedded in glass slides were collected and hematoxylin and eosin (HE) and immunohistochemical staining were performed. Microscopic characteristics, including cell morphology, cell density, mitotic rate, nuclear atypia, necrosis, and vascular invasion, were examined using HE staining. To assess malignancy potential, the classification reported by Folpe et al. was used. Immunohistochemical staining for cytokeratin (clone AE1&AE3; Leica Biosystems, UK), monoclonal anti-episialin (EMA, clone GP1.4; Leica Biosystems), vimentin (clone V9; Leica Biosystems), S-100 (Leica Biosystems), desmin (clone DE-R-11; Leica Biosystems), Smooth Muscle Actin (SMA, clone α sm-1; Leica Biosystems), Muscle-Specific Actin (MSA, clone HHF35; Leica Biosystems), CD34 (clone QBEnd/10; Leica Biosystems), HMB45 (clone HMB45; Leica Biosystems), Melan A (clone A103; Leica Biosystems), melanoma (clone PNL2, dilution 1:50; Dako, CA, USA), Ki67 (clone MIB-1, dilution 1:100; Dako), and p53 (clone DO-7; Leica Biosystems) was performed using the BOND III Fully Automated IHC and ISH Stainer (Leica Biosystems, Germany). Immunohistochemical staining for estrogen receptor (ER, clone SP1; Ventana Medical Systems, AZ, USA), progesterone receptor (PR, clone 1E2; Ventana Medical Systems), SOX-10 (clone SP267; Roche), BRAF V600E (clone VE1; Ventana Medical Systems) and Transcription factor binding to IGHM enhancer 3 (TFE3, clone MRQ-37; Ventana Medical Systems) was performed using the Ventana BenchMark ULTRA automated immunostainer (Ventana Medical Systems). The slides were observed under an optical microscope and evaluated by two pathologists specialized in bone and soft tissue oncology (IY and FM). The percentage of positive cells was classified as negative, 5–25%: (1 +), 26–50%: (2 +), and > 51%: (3 +).

### Statistical analysis

Statistical analysis was conducted using the Bell Curve for Excel (Social Survey Research Information Co., Ltd.). Survival analysis was performed using the Kaplan–Meier method. Overall survival was defined as the time from diagnosis to the final investigation.

## Results

### Clinical characteristics

**Fig. 1:**
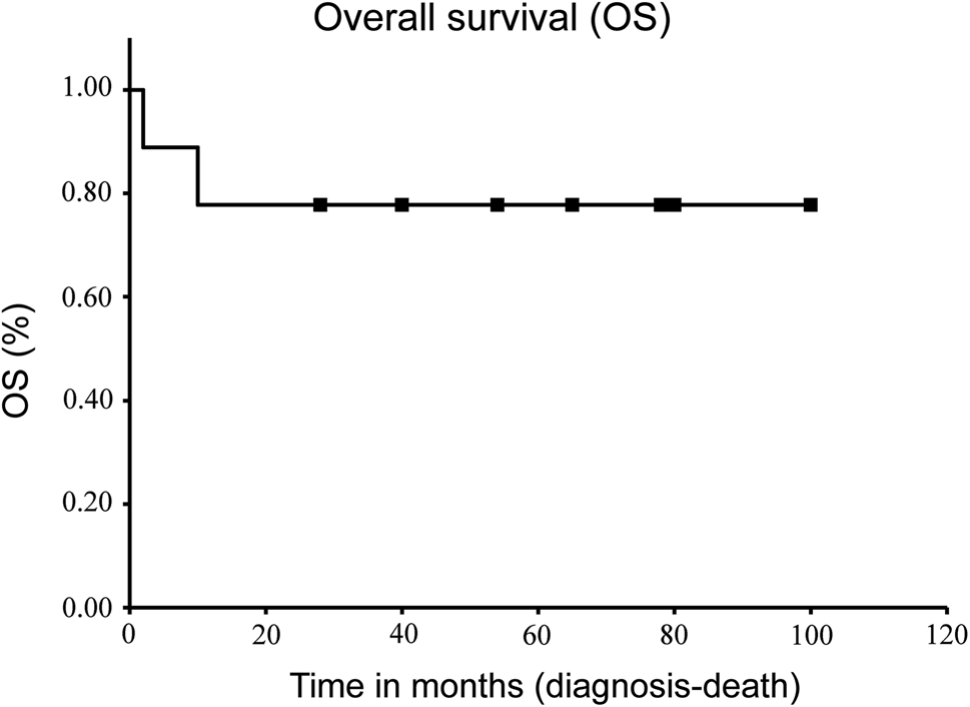
Kaplan–Meier curves for overall survival. The 1-year overall survival rate was 77%

The patient details are presented in Table 1. There were four males and five females with a median age of 50 years (46–83 years). There were eight cases of soft tissue development and one case of bone development. Tumor locations were the upper extremities in three, lower extremities in three, and trunk in three patients. The mean tumor diameter was 8.8 cm (1.8–20.2 cm); the diameters of tumors were > 5 cm in five cases (56%). Two patients had lung metastasis at the time of initial treatment (cases 3 and 6). Surgery was performed in nine cases. Chemotherapy was administered to one patient with advanced disease (case 4) (1st line: 2 courses of doxorubicin and ifosfamide, 2nd line: a course of ifosfamide, carboplatin and etoposide, 3rd line: pazopanib), and radiotherapy was administered in two patients, one of which was for local recurrence (case 2) (heavy ion therapy with the total dose of 70.4 Gy/16 fractions), and the other was for advanced disease (case 3) (the total dose of 40 Gy/16 fractions). The mean follow-up period was 50.8 months (2–100 months), and the 1-year survival rate was 78% (Fig. 1). One patient (case 4) had postoperative distant metastasis to the lungs and common iliac lymph nodes. One patient (case 2) experienced local recurrence 8 months after surgery and received radiation therapy. Thereafter, the patient developed postoperative distant metastasis to the lungs four years after the initial therapy and underwent pulmonary metastasectomy. The oncological outcomes at the final follow-up period were continuous disease-free status in six cases, no evidence of disease in one case, alive with disease in one case, and dead of disease in two cases.

**Table 1:** Details of patient characteristics

No	Age	Gender	Origin	Location	Tumor size (cm)	Treatment	Local recurrence	Distant metastasis	Follow-up period (months)	Outcome
1	46	F	S	Thigh	4.0	Surgery	–	–	65	CDF
2	47	F	B	Lumbar	5.0	Surgery and radiation	+	+	70	NED
3	64	M	S	Axillary	20.2	Radiation	–	+	2	DOD
4	48	M	S	Thigh	18.0	Surgery and chemotherapy	–	+	10	DOD
5	47	M	S	Back	4.5	Surgery	–	–	28	CDF
6	77	F	S	Thigh	4.3	Surgery	–	+	54	AWD
7	59	F	S	Chest wall^a^	6.7	Surgery	–	–	100	CDF
8	83	M	S	Axillary	15.0	Surgery	–	–	51	CDF
9	50	F	S	Elbow	1.8	Surgery	–	–	78	CDF

*M* male, *F* female, *S* soft tissue, *B* bone, *CDF* continuous disease-free, *NED* no evidence of disease, *AWD* alive with disease, *DOD* dead of disease

^a^Located in the musculus pectoralis major

### Histopathological findings

Details of the pathological features are shown in Table 2A. Most cases showed a nested architecture, comprising epithelioid cells with abundant granular eosinophilic or clear cytoplasm as well as round nuclei with small nucleoli. The tumor cells were arranged radially around the blood vessels. Morphological findings included six tumors with predominantly epithelioid cells, two tumors with predominantly spindle cells, and one tumor with equal proportions of epithelial and spindle cells. Clear peritumoral borders were observed in eight cases, and one case was borderline invasive, which led to local recurrence. Tumors with nuclear atypia were observed in eight cases (89%), with high mitotic rates in eight cases (89%), with high cell density in eight cases (89%), and with necrosis in five cases (56%). Tumor vascular invasion was observed in two cases (22%) and led to local recurrence in one case and distant metastasis in the other. Eight cases (89%) were classified as malignant, and one case (11%) was classified as having uncertain malignancy potential (Table 2A). All four cases with local recurrence or distant metastases were classified as tumors with malignancy potential. None of the patients were classified as benign.

**Table 2:** Pathological features (A) and immunohistochemical analysis results (B)

(A)									
No.	Morphology	Size > 5 cm	Infiltrative	Nuclear grade	High cellularity	Mitotic rate ≧1/50 HPF	Necrosis	Vascular invasion	Folpe classification
**1**	Epithelioid	–	–	+	–	–	–	–	U
**2**	Epithelioid	+	+	+	+	+	+	+	M
**3**	Spindle	+	–	–	+	+	+	–	M
**4**	Mixed	+	–	+	+	+	+	–	M
**5**	Spindle	–	–	+	+	+	–	–	M
**6**	Epithelioid	–	–	+	+	+	–	+	M
**7**	Epithelioid	+	–	+	+	+	+	–	M
**8**	Epithelioid	+	–	+	+	+	+	–	M
**9**	Epithelioid	–	–	+	+	+	–	–	M

(B)																		
No.	CK	EMA	Vim	S–100	Desmin	SMA	MSA	CD34	HMB45	Melan A	Melanoma	ER	PR	p53	TFE3	BRAF V600E	SOX–10	MIB–1 index
1	–	–	2 +	–	–	–	–	–	1 +	–	1 +	–	2 +	3 +	1 +	–	–	5%
2	–	–	2 +	–	–	–	–	–	1 +	2 +	2 +	–	–	2 +	2 +	–	–	50%
3	–	–	3 +	–	–	–	–	–	–	–	–	–	–	–	2 +	–	–	1%
4	1 +	1 +	3 +	–	2 +	1 +	1 +	–	1 +	–	1 +	–	2 +	3 +	3 +	–	–	50%
5	–	–	3 +	–	–	3 +	3 +	–	1 +	–	1 +	3 +	1 +	1 +	3 +	–	–	5%
6	–	3 +	–	–	–	2 +	–	–	1 +	2 +	2 +	–	–	2 +	2 +	–	–	30%
7	–	1 +	3 +	–	–	–	–	–	2 +	–	2 +	–	–	–	3 +	–	–	5%
8	–	2 +	2 +	–	–	–	–	–	1 +	–	1 +	–	1 +	1 +	2 +	–	–	60%
9	–	–	–	–	1 +	1 +	N.A	–	1 +	1 +	N.A	N.A	N.A	N.A	N.A	N.A	N.A	60%
Summary	11% (1/9)	44% (4/9)	78% (7/9)	0% (0/9)	22% (2/9)	44% (4/9)	25% (2/8)	0% (0/9)	89% (8/9)	33% (3/9)	88% (7/8)	13% (1/8)	50% (4/8)	75% (6/8)	100% (8/8)	0% (0/8)	0% (0/8)	

*U* uncertain malignant potential, *M* malignant, *CK* cytokeratin, *Vim* vimentin, *SMA* smooth muscle actin, *MSA* muscle-specific actin, *ER* estrogen receptor, *PR* progesterone receptor, *TFE3* transcription factor binding to IGHM enhancer 3, *N.A* not available

The percentage of positive cells was classified as negative, 5–25%: (1 +), 26–50%: (2 +), and > 51%: (3 +)

### Immunohistochemical findings

**Fig. 2:**
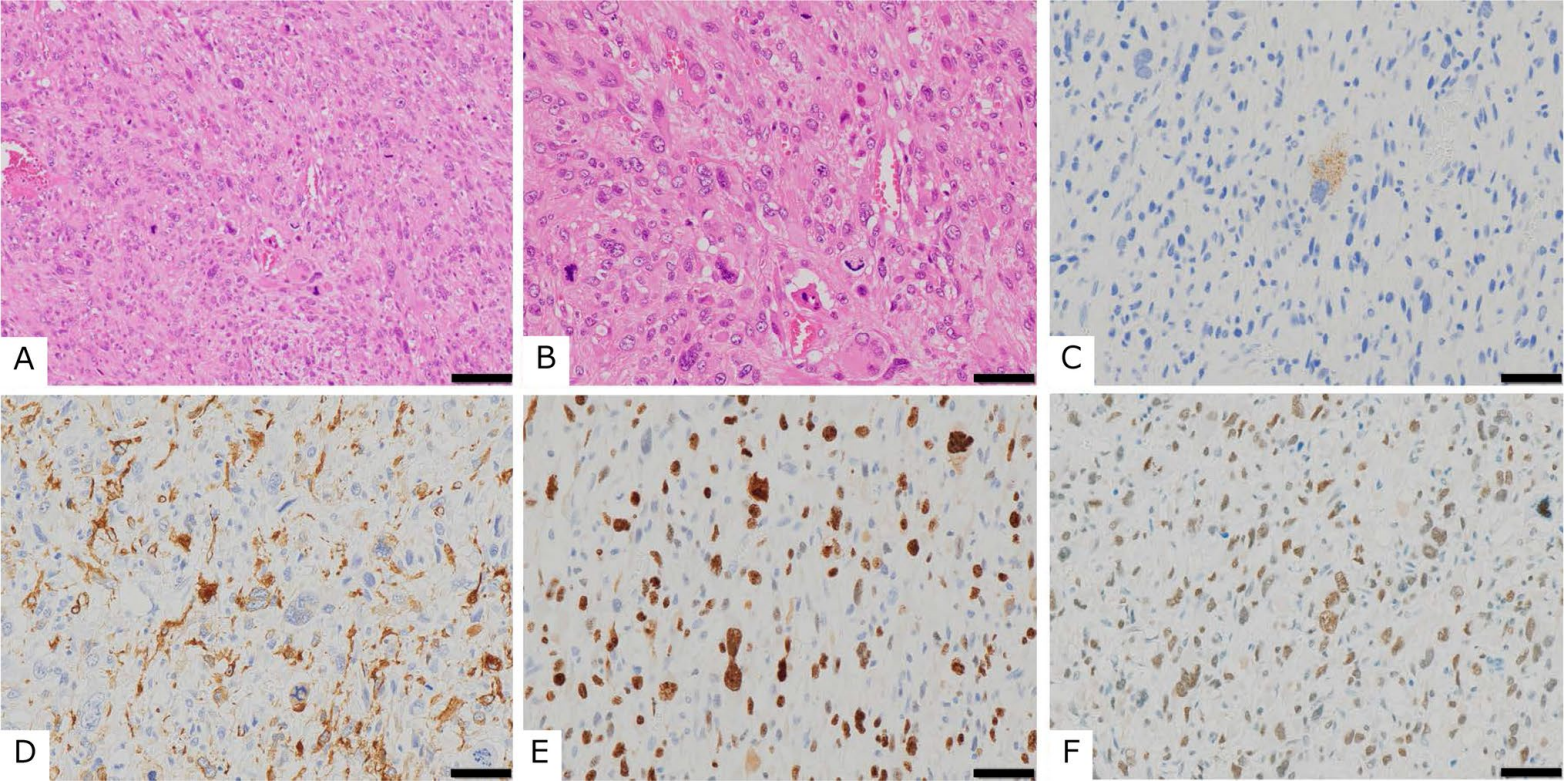
Morphological and immunohistochemical appearance of case no. 4 (Table 2). **A** Low-power and **B** high-power images of hematoxylin and eosin staining. Immunohistochemical findings revealed positive staining for **C** Melanoma, **D** SMA, **E** MIB-1, and **F** TFE3. Scale bars: 100 µm (**A**) and 50 µm (**B**–**F**)

The results of immunohistochemical staining are presented in Table 2B. Eight of nine tumors (89%) were positive for HMB45, and 7 of 8 (88%) were positive for melanoma antibodies, indicating a high positivity rate for melanocytic markers. In contrast, a relatively lower expression of muscle marker proteins was observed. Moreover, 4/9 (44%) tumors were positive for SMA, 2/8 (25%) for MSA, and 2/9 (22%) for desmin. The positivity rate of muscular markers was lower than that of melanocyte markers. Because expression of SOX-10 and BRAF V600E were negative in all cases, melanoma could be excluded. TFE3 expression was positive in 100% (8/8) of the evaluable cases, and three of these cases showed strong (3 +) nuclear staining for TFE3 (Table 2B; cases 4, 5, and 7). Tumors with high expression of TFE3 (2 + or higher) were classified as PEComas with malignant potential according to Folpe’s classification. 5 of 9 cases (56%) showed high MIB-1 labeling index values of more than 30%. We showed an example of the typical morphological and immunohistochemical findings in Fig. 2.

## Discussion

PEComas of the bone and soft tissues are extremely rare. In the current study, we examined the clinical and pathological characteristics of PEComas of bone and soft tissues (Tables 1 and 2) and compared them with the characteristics of PEComas of various organs, including the skin, uterus, gastrointestinal tract, pancreas, kidney, liver, bone, and soft tissue (Tables 3 and 4) (Mahera et al. 1997; Kuroda et al. 2000; Diment and Colecchia 2003; Fukunaga 2004; Harris et al. 2004; Folpe et al. 2005; Mai and Belanger 2006; Blechet et al. 2007; Pikoulis et al. 2007; Osei et al. 2007; Boussouga et al. 2008; Argani et al. 2010; Varshney et al. 2011; Charli-Joseph et al. 2014; Conlon et al. 2015; Chen et al. 2016; Zhang et al. 2017, 2021; Alnajar et al. 2018; Bao et al. 2019; Harvey et al. 2019; Zhong et al. 2020; Rehman et al. 2021).

**Table 3:** Previous reports of soft-tissue PEComa

No	Authors	Publication year	Age	Gender	Location	Size (cm)	Melan-A	HMB45	SMA	Desmin	CK	CD34	S-100	TFE3
1	Mahera et al.	1997	79	F	Leg	21	N.A	-	+	+	NA	+	NA	NA
2	Kuroda et al.	2000	41	M	Thigh	10	N.A	+	N.A	NA	NA	NA	NA	NA
3	Diment and Colecchia.	2003	59	F	Thigh	10	N.A	+	+	–	–	NA	–	NA
4	Fukunaga.	2004	44	F	Abdominal wall	3.5	+	+	+ +	–	+	–	–	NA
5	Harris et al.	2004	87	M	Knee	5.5	N.A	+	+	–	–	–	–	NA
6	Folpe et al.	2005	43	F	Thigh	3.5	–	+	+	–	NA	NA	–	NA
7			71	M	Forearm	9	N.A	N.A	N.A	N.A	N.A	N.A	N.A	NA
8			77	F	Neck	2.6	N.A	N.A	N.A	N.A	N.A	N.A	N.A	NA
9			49	F	Shoulder	Large	N.A	N.A	N.A	N.A	N.A	N.A	N.A	NA
10			22	F	Abdominal wall	8.5	N.A	N.A	N.A	N.A	N.A	N.A	N.A	NA
11			24	F	Abdominal wall	10.5	N.A	N.A	N.A	N.A	N.A	N.A	N.A	NA
12			19	F	Pelvic soft tissue	2.1	N.A	N.A	N.A	N.A	N.A	N.A	N.A	NA
13			18	F	Pelvic soft tissue	6	N.A	N.A	N.A	N.A	N.A	N.A	N.A	NA
14			72	F	Pelvic soft tissue	24	N.A	N.A	N.A	N.A	N.A	N.A	N.A	NA
15	Mai and Belanger	2006	56	M	Thigh	11	N.A	+ +	N.A	+ +	+	NA	–	NA
16			46	M	Groin	8	N.A	+ +	N.A	–	–	NA	–	NA
17			60	F	Thigh	12	N.A	+	N.A	+	+	NA	–	NA
18	Pikoulis et al.	2007	23	F	Buttock	8.5	N.A	–	+ +	NA	NA	NA	NA	NA
19	Osei et al.	2007	49	F	Shoulder	5.3	N.A	+	+	NA	NA	N.A	-	NA
20	Blechet et al.	2007	13	M	Knee Joint	4	N.A	+	+	NA	NA	NA	-	NA
21	Boussouga et al.	2008	26	F	Shoulder	6	N.A	+	+	+	NA	NA	NA	NA
22	Argani et al.	2010	33	F	Buttock	N.A	–	+	–	–	–	–	–	+ +
23			46	F	Thigh	6.5	+	+	–	–	–	–	–	+ +
24			71	M	Arm	4	–	+	–	+	–	N.A	–	+ +
25			26	F	Thigh	3	+	N.A	–	N.A	N.A	N.A	N.A	+
26			48	F	Foot	N.A	+	+	+	+	N.A	N.A	N.A	+
27	Varshney et al.	2011	12	F	Knee joint, foot	15	N.A	–	+	+	NA	NA	NA	NA
28	Alnajar et al.	2017	44	M	Knee	23	–	+	+	NA	–	NA	–	–
29	Harvey et al.	2019	44	M	Knee	6.5	+	–	+	–	N.A	N.A	–	–
30	Rehman et al.	2021	49	F	Buttock	10.5	N.A	+	+	–	N.A	–	–	NA
Summary of previous reports		1997–2021	45	F/M: 21/9	Upper extremity: 5 Lower extremity: 15 Trunk: 10	8.9	56% (5/9)	81% (17/21)	78% (14/18)	44% (7/16)	30% (3/10)	17% (1/6)	0% (0/15)	71% (5/7)
Present study		2023	50	F/M: 5/4	Upper extremity: 3 Lower extremity: 3 Trunk: 3	8.8	33% (3/9)	89% (8/9)	44% (4/9)	22% (2/9)	11% (1/9)	0% (0/9)	0% (0/9)	100% (8/8)

**Table 4:** Comparison of immunohistochemical findings among various PEComa origins

Authors	Publication year	Primary origin (no. of patients)	Age (median)	Gender F/M	Melan-A	HMB45	SMA	Desmin	CK	CD34	S-100	TFE3	High MIB-1 index (> 10%)
Charli-Joseph et al.	2014	Skin (8)	46	5/3	0% (0/8)	88% (7/8)	88% (7/8)	13% (1/8)	NA	NA	38% (3/8)	NA	13% (1/8)
Conlon et al.	2015	Uterine corpus (78)	47.5	78/0	46% (21/46)	99% (71/72)	80% (53/68)	63% (39/62)	5% (2/43)	0%	NA	NA	NA
Chen et al.	2016	GI-tract (26)	38.9	32/18	65% (22/34)	96% (44/46)	64% (31/33)	44% (18/44)	0% (0/21)	0% (0/32)	11% (4/37)	60% (6/10)	NA
Zhang et al.	2017	Pancreas (21)	47.9	17/3	NA	100% (20/20)	88% (15/17)	NA	6% (1/18)	NA	NA	NA	0% (0/12)
Bao et al.	2019	Kidney (24) and others (2)	51	22/4	100% (26/26)	96% (25/26)	100% (26/26)	85% (22/26)	0% (0/26)	0% (0/26)	27% (7/26)	0% (0/26)	NA
Zhang et al.	2021	Liver (26)	50	17/9	88% (23/26)	96% (25/26)	84% (22/26)	4% (1/26)	NA	69% (18/26)	54% (14/26)	0% (0/26)	4% (1/26)
Zhong et al.	2020	Bone (20)	37	10/10	50% (10/20)	80% (16/20)	45% (9/20)	15% (3/20)	NA	NA	5% (1/20)	4cases	NA
Summary of previous reports^a^	1997–2021	Soft tissue (30)	45	21/9	56% (5/9)	81% (17/21)	78% (14/18)	44% (7/16)	30% (3/10)	17% (1/6)	0% (0/15)	71% (5/7)	NA
Present study	2023	Bone (1) and soft tissue (8)	50	5/4	33% (3/9)	89% (8/9)	44% (4/9)	22% (2/9)	11% (1/9)	0% (0/9)	0% (0/9)	100% (8/8)	56% (5/9)

GI-tract: gastrointestinal tract, F: female, M: male

^a^Summary of previous reports was obtained from Table 3

The present study included four males and five females with a median age of 50 years (4683 years). Bone and soft tissue PEComas are more frequent in females than in males and usually occur in middle or old age. In terms of age and sex, PEComas of the bone and soft tissue showed a similar trend to that reported in previous studies of PEComas arising in other organs (Table 4). Bone and soft tissue PEComas occurred almost equally in the upper extremities, lower extremities, and trunk, and there was no specific site of predilection. The mean tumor diameter was 8.8 cm, and tumors were larger than 5 cm in five cases. The PEComas of the bone and soft tissue were usually large at the time of detection. These findings are comparable to those of previously reported soft tissue PEComas (Table 3).

There have only been a few detailed reports on the clinical prognosis of PEComas. Conlon et al. reviewed 78 cases of uterine corpus PEComas and reported that ten of the 63 patients (16%) died of the disease; the median survival of these ten patients was 20 months. They also reported that nine patients (14%) were alive with disease, while 44 (70%) had no evidence of disease (Conlon et al. 2015). In the present study, two patients had lung metastasis at the time of initial treatment (cases 3 and 6). One patient had postoperative distant metastasis to the lung and common iliac lymph nodes (case 4). One patient experienced local recurrence 8 months after surgery and received radiation therapy. Thereafter, the patient developed postoperative distant metastasis to the lung 4 years after the initial therapy and underwent pulmonary metastasectomy (case 2). The 1-year survival rate was 78% (Fig. 1). The prognosis of bone and soft tissue PEComas is comparable to that of the uterine corpus (Conlon et al. 2015). Although it was difficult to conclude the clinical risk of developing bone and soft tissue PEComas due to the short follow-up period of the current study, bone and soft tissue PEComas were found to have a poor prognosis, similar to that of high-grade soft tissue sarcomas.

PEComas typically show a nested architecture, comprising epithelioid cells with abundant granular eosinophilic or clear cytoplasm, round nuclei, and small nucleoli. Nests or trabeculae are typically surrounded by thin-walled capillary vessels. In contrast, a small subset of PEComas has predominantly spindle cell morphology (Bonetti et al. 2001). In the bone and soft tissue PEComas, the morphological findings included six tumors with predominantly epithelioid cells, two tumors with predominantly spindle cells, and one tumor with equal proportions of epithelioid and spindle cells. Malignant PEComas are typically characterized by large tumor size, mitoses, necrosis, and nuclear atypia. Folpe et al. developed a prognostic system based on the retrospective analysis of 26 PEComas at multiple sites and divided them into benign, uncertain malignancy potential, and malignancy groups based on histological criteria (Folpe et al. 2005). Based on this classification, malignancy was reported in 57% (12/21 cases) of PEComas of the bone (Zhong et al. 2020), 54% (42/78 cases) of uterine PEComas (Conlon et al. 2015), and 52% (26/50 cases) of gastrointestinal PEComas (Chen et al. 2016). In the study of soft tissues by Folpe et al., excluding those of uterine, intra-abdominal, and genital origin, seven cases were classified as malignant, one case had uncertain malignancy potential, and one case was benign (cases 6–14) (Folpe et al. 2005). In the present study, 89% of the bone and soft tissue PEComas were classified as malignant, while 11% were classified as having uncertain malignancy potential. None of the patients were categorized as benign. More than half of the cases (5/9) showed high MIB-1 index values of > 10% (Table 2B). In contrast, the rates of occurrence of high MIB-1 index values (> 10%) were 13% (1/8) in skin PEComa, 0% (0/12) in pancreatic PEComa, and 4% (1/26) in liver PEComa (Charli-Joseph et al. 2014; Zhang et al. 2017, 2021). These results support the hypothesis that bone and soft tissue PEComas have a particularly higher cell proliferation and malignancy potential than other visceral PEComas (Hasegawa et al. 2002).

PEComa is characterized by the immunohistochemical expression of both melanocytic and muscle markers (Utpatel et al. 2020). In this study, the melanocyte marker HMB45, which is considered the most sensitive immunostaining marker, showed a high positivity rate of 89%. Melanoma also showed a high positivity rate of 88%. Because expression of SOX-10 and BRAF V600E were negative in all cases, melanoma could be excluded (Miettinen et al. 2015; Mohamed et al. 2013). Meanwhile, the muscle markers SMA, MSA and desmin were positive only in 44%, 25%, and 22% cases, respectively. Muscle markers were less abundant than melanocytic markers and tended to be less abundant than those in PEComas of other primary sites (Table 4) (Charli-Joseph et al. 2014; Conlon et al. 2015; Chen et al. 2016; Zhang et al. 2017, 2021; Bao et al. 2019; Zhong et al. 2020; Rehman et al. 2021). In general, epithelioid PEComas tend to show higher expression of melanocytic markers than that of myogenic markers, and spindle cell PEComas show an opposite expression profile (Conlon et al. 2015). In this study, 67% of the cases showed epithelioid and 33% showed spindle cell morphology or mixed morphology. This may have resulted in the high expression of melanocytic markers and low expression of myogenic markers in the bone and soft tissue PEComa.

PEComas are classified into two subtypes. Conventional PEComas harbor mutations and loss of heterozygosity (LOH) in the *TSC2* gene and, more rarely, the *TSC1* gene, which may be associated with angiomyolipomas and PEComas. The significance of LOH in TSC1/2 is the subsequent upregulation of mTOR signaling, which is the basis of the action of mTOR inhibitors that are often utilized in PEComa treatment. In contrast, a distinct small subset of PEComas harboring rearrangements of the TFE3(Xp11) gene locus has been identified (Utpatel et al. 2020). The TFE3-rearranged PEComas harbor *TFE3* gene fusions, which correlate with a strong nuclear immunoreactivity for TFE3 (Argani et al. 2010; Malinowska et al. 2012); approximately 15% cases show strong nuclear staining for TFE3 (Bonetti et al. 2001). These tumors tend to have a prominent alveolar pattern and epithelioid morphology and lack the expression of smooth muscle markers (Malinowska et al. 2012; Utpatel et al. 2020). In our study, TFE3 expression was positive in 100% cases that could be observed; three of these cases showed strong (3 +) nuclear staining for TFE3 (Table 2B; cases 4, 5, and 7). In addition, tumors with high expression of TFE3 (2 or higher) (cases 2–8) were classified as PEComas with malignant potential according to Folpe’s classification. These characteristics suggest that PEComas of the bone and soft tissue are prone to *TFE3* rearrangement with high malignant potential.

Radical resection is the primary treatment modality for PEComas, as PEComas are characterized by resistance to radiation and chemotherapy (Bleeker et al. 2012; Jia et al. 2020). Radical resection is associated with an increased disease-free survival (Sobiborowicz et al 2021). Recently, mTOR inhibitors such as sirolimus have been shown to be effective for inoperable, recurrent, or advanced PEComas (Switaj et al. 2021). In contrast to conventional PEComas, TFE3-rearranged PEComas were shown to lack *TSC2* inactivating mutations (Switaj et al. 2021). These findings have theoretically critical treatment implications, particularly for the efficacy of targeted mTOR inhibitors, as the hypothetical benefit of this therapy is likely minimized. Therefore, recognition of the rearranged variant of PEComa may assist in making important decisions regarding clinical management (Schoolmeester et al. 2015). Our study shows that PEComas of the bone and soft tissues are more likely to develop into TFE3-rearranged PEComas, which may be useful for developing future treatment strategies.

The present study has several limitations. The first serious limitation was that this study included only a small number of cases. Because PEComas of bone and soft tissue tumors are extremely rare, an international study is necessary to collect more information. Second, we could not genetically identify TSC1/TSC2 alterations or *TFE3* gene rearrangements. Third, we excluded primary PEComa of the visceral organs, such as the gastrointestinal tract, uterus, and kidney, from our analysis. Thus, we could not compare the chronological characteristics or histological details of PEComas of the bone and soft tissue with those of the other visceral organs. Further genetic investigations involving a larger number of patients are necessary to establish an appropriate treatment strategy for PEComas.

## Conclusions

Our study indicated that bone and soft tissue PEComas may have a particularly higher malignancy potential than other visceral PEComas. Bone and soft tissue PEComas are more likely to result in TFE3 rearrangements. Further studies, combined with genetic and molecular exploration, are required.

## Data Availability

The data that support the findings of this study are available from the corresponding author upon reasonable request.
